# iTRAQ proteomic analysis of extracellular matrix remodeling in aortic valve disease

**DOI:** 10.1038/srep17290

**Published:** 2015-12-01

**Authors:** Tatiana Martin-Rojas, Laura Mourino-Alvarez, Sergio Alonso-Orgaz, Esther Rosello-Lleti, Enrique Calvo, Luis Fernando Lopez-Almodovar, Miguel Rivera, Luis R. Padial, Juan Antonio Lopez, Fernando de la Cuesta, Maria G. Barderas

**Affiliations:** 1 Department of Vascular Physiopathology, Hospital Nacional de Parapléjicos, SESCAM, Toledo, Spain; 2 Cardiocirculatory Unit, Health Research Institute, Hospital La Fe, Valencia, Spain; 3 Proteomics Core Facility, CNIC, Madrid, Spain; 4 Cardiac Surgery, Hospital Virgen de la Salud, SESCAM, Toledo, Spain; 5 Department of Cardiology, Hospital Virgen de la Salud, SESCAM, Toledo, Spain

## Abstract

Degenerative aortic stenosis (AS) is the most common worldwide cause of valve replacement. The aortic valve is a thin, complex, layered connective tissue with compartmentalized extracellular matrix (ECM) produced by specialized cell types, which directs blood flow in one direction through the heart. There is evidence suggesting remodeling of such ECM during aortic stenosis development. Thus, a better characterization of the role of ECM proteins in this disease would increase our understanding of the underlying molecular mechanisms. Aortic valve samples were collected from 18 patients which underwent aortic valve replacement (50% males, mean age of 74 years) and 18 normal control valves were obtained from necropsies (40% males, mean age of 69 years). The proteome of the samples was analyzed by 2D-LC MS/MS iTRAQ methodology. The results showed an altered expression of 13 ECM proteins of which 3 (biglycan, periostin, prolargin) were validated by Western blotting and/or SRM analyses. These findings are substantiated by our previous results demonstrating differential ECM protein expression. The present study has demonstrated a differential ECM protein pattern in individuals with AS, therefore supporting previous evidence of a dynamic ECM remodeling in human aortic valves during AS development.

With a growing older population, cardiovascular diseases are becoming an increasing economic and social burden in Western societies. Cardiovascular calcification is a major characteristic of chronic inflammatory disorders such as chronic renal disease, atherosclerosis and calcific aortic valve disease, which are associated with significant morbidity and mortality^1^. Currently, degenerative aortic stenosis (AS) is the main cause of aortic valve replacement in developed countries. It is characterized by a narrowing of the aortic valve opening which causes the obstruction of the blood flow from the left ventricle into the aorta^2^. With all this in mind, it is important to note that the ability of the valve to open and close repeatedly, as well as the maintenance of the phenotypes of valvular cells, is made possible by the tissue microstructure. This microstructure is influenced by the composition and orientation of the extracellular matrix (ECM). The ECM within the aortic valve is a dynamic environment rich in collagen, elastic fibers, proteoglycans and glycosaminoglycans, growth factors, proteases, signaling molecules and secreted proteins^3^. The ECM provides an organized framework which enforces cell orientation and structural integrity, allowing valve ability. Indeed, ECM is involved in valvular remodeling, which is typically described as a process composed of three main overlapping phases: inflammatory, proliferative and maturation^4^,^5^. Despite the growing evidence connecting ECM remodeling with AS pathogenesis, the underlying regulatory mechanisms for such remodeling and stratification are not well defined, even though they seem relevant to interpret aortic valve degeneration. In previous studies in our lab^6^, we conducted an in-depth analysis of the secretome of aortic valves which pointed out an outstanding role of valvular remodeling in the pathophysiological mechanisms of AS. The functional classification of the differentially secreted proteins showed a significant number of ECM proteins. Since many ECM proteins are involved in AS, and taking into account its high prevalence and the long course of this disease, a better understanding of AS at the molecular level may be possible through a proteomic approach. This powerful technique might provide useful information to better understand the development of the disease. Besides, it may offer a new gate for the discovery of novel potential biomarkers shed to blood by the aortic valve, which may allow detecting patients at risk of developing degenerative AS, as a starting point.

In fact, previous studies of our group using two-dimensional difference in-gel electrophoresis (2D-DIGE) showed several ECM proteins altered with the progression of AS.^6^,^7^. According to our background, the aim of this work is to further clarify AS process and to ascertain whether ECM proteins might play a determinant role in AS. Here, we compared human AS valve tissue with that of healthy subjects using isobaric tags for relative and absolute quantitation (iTRAQ), a robust technique which presents more sensitivity and better accuracy of quantification than 2D-DIGE^8^ aiming to complement previous analyses with AS tissue using a deeper approach. Validation of 3 ECM proteins found altered was performed by orthogonal techniques (Western blotting and/or selected reaction monitoring (SRM)).

## Results

### Quantitative proteomics: iTRAQ labeling

To provide insight into the consequences of AS on protein expression, we compared the proteome of AS tissue with the one from healthy valves. Lysates from 4 control samples and 4 stenotic samples were iTRAQ labeled as shown in Fig. 1 of supplementary material (Figure SM1). Samples were pooled in four labeled peptide mixtures to allow the analysis of all of them in a limited number of LC-MS/MS runs avoiding labeling bias (for experimental design see supplementary material). After the labeling procedure and liquid chromatography (LC) separation, derived peptides were identified and quantified by tandem mass spectrometry (MS/MS). The data generated by liquid chromatography coupled to mass spectrometry (LC-MS/MS) analysis were searched against the human RefSeq database using Proteome Discoverer software (Thermo Scientific).

**Figure 1:**
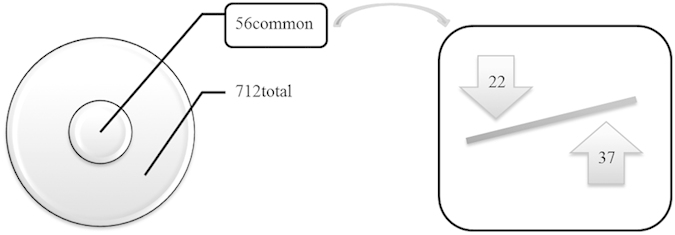
Schematic representation of the proteins identified in the iTRAQ experiment. Within the 712 identified proteins, 56 proteins (34 up-regulated and 19 down-regulated in stenotic aortic valves when compared to controls, in addition to 3 proteins which are increased or decreased depending on the isoform identified in stenotic valves when compared to controls) were detected in the four runs (mixtures) of the experiment carried out.

iTRAQ ratios corresponding to 712 proteins between control and stenotic samples in four separate experiments were compared (Fig. 1).

**Figure 2:**
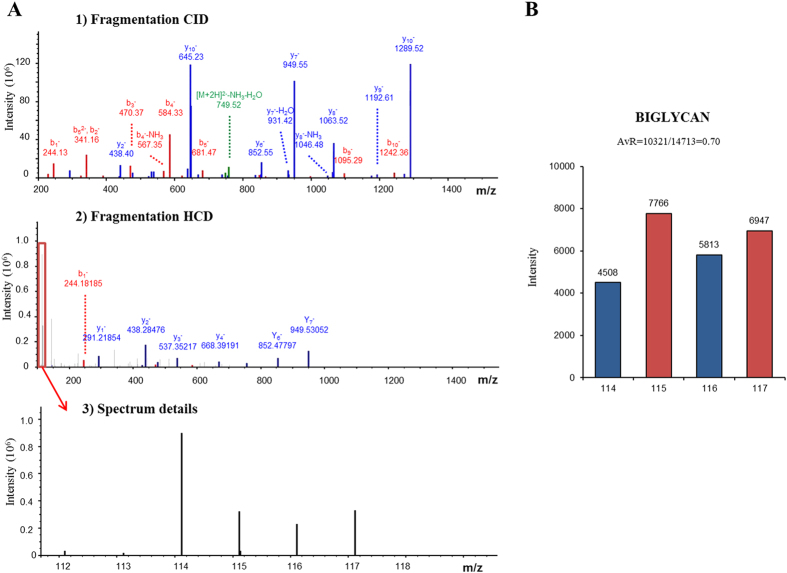
(**A**) Example of one of the proteins identified after iTRAQ labeling and LC-MS/MS analysis. This protein was up-regulated in aortic stenosis (labeled with 115 and 117), with respect to control valves (labeled with 114 and 116). (1) MS/MS spectra after collision-induced dissociation (CID), (2) MS/MS fragmentation spectra after high-energy collision dissociation (HCD) for the analysis of low molecular masses, (3) detail of low molecular weight area after HCD fragmentation, showing the peaks that correspond to the reporter ions. (**B**) Quantification of reporter ion intensities for one of the proteins varied after iTRAQ label, biglycan. As can be observed, the ratio (114 + 116)/(115 + 117), in this case corresponding to controls/AS, is lower than the 0.71 cutoff, which means this protein is increased in AS.

For each experiment, we examined the original protein list generated by Protein Discoverer and filtered it according to the following criteria: a manual inspection of spectra was carried out wherever identification was supported among 90−95% confidence values. Fold changes were calculated by comparing iTRAQ reporter ion intensities of peptides derived from AS and control valves (representative peptide MS/MS spectra and reporter ions of some differentially expressed proteins are shown in Fig. 2). Using 1.4-0.71 fold ratio as a cutoff to assign up or down-regulated proteins, we observed 56 differential proteins present in the four runs (mixtures) of the experiment, 34 up-regulated and 19 down-regulated in stenotic aortic valves, in addition to 3 proteins (haptoglobin, peptidyl-prolyl cis trans isomerase A, L-lactate dehydrogenase A chain isoform 1) which were increased or decreased depending on the isoform identified (Tables 1 and 2).

### Annotation and functional enrichment

**Figure 3:**
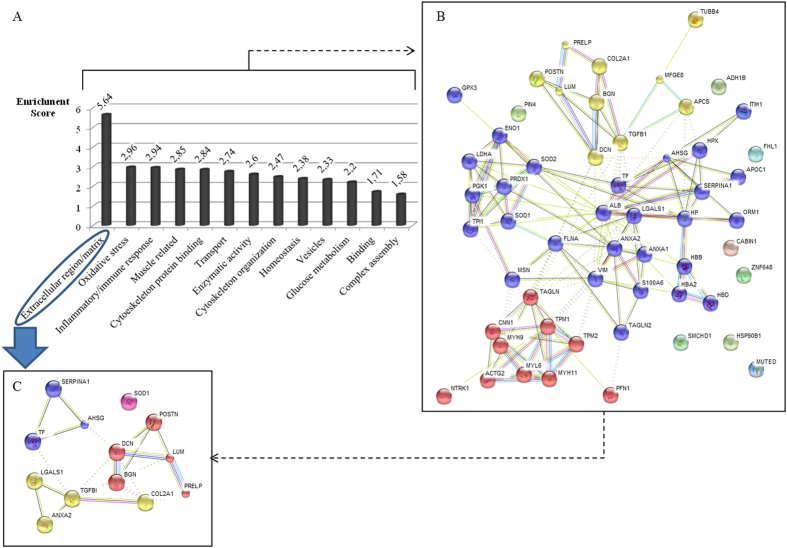
(**A**) Biological implications of the 56 differentially expressed proteins according to the functional annotation clustering. (**B**,**C**) Protein-protein interaction networks were studied using the STRING v9.1 web-tool. Analyses of the 56 dysregulated proteins (**B**) and ECM proteins (**C**) are shown. Proteins with the same circle colours correspond to the same cluster, according to the classification performed using this tool.

Functional analysis of the altered proteins was performed using DAVID v6.7. Molecular functions and biological processes were explored through the functional annotation tool to generate clusters of overrepresented Gene Ontology (GO) terms. This analysis showed 13 significant clusters where the most significant cluster was formed by proteins from the extracellular region or matrix. This cluster is composed of 24 proteins of which 13 correspond to ECM proteins. Among most significantly enriched terms we observed oxidative stress, inflammatory response and proteins related to muscle and cytoskeleton. ECM proteins together with oxidative stress proteins are primarily involved in the biological AS process (Fig. 3A).

### Protein-protein interaction

The 56 deregulated proteins determined in the iTRAQ experiment were introduced into the web-tool STRING v9.1 to generate protein-protein interaction networks (Fig. 3B). We selected exclusively the 13 ECM proteins which appeared altered in the iTRAQ experiment attending to the functional clustering and we analyzed them by web-tool STRING v9.1 too. (Fig. 3C).

After the clustering of ECM proteins, we found that the biggest group was composed by 5 proteins, all of which are tightly connected to each other (Fig. 3C, red cluster). Among these differentially expressed proteins were biglycan (BGN) with a control/patient ratio of 0.70, periostin (POSTN) with a ratio of 0.55 and prolargin (PRELP) with a ratio of 0.59. These 3 proteins were identified in the four iTRAQ experiments (mixtures) and were selected for the validation of the quantitative proteomic analysis.

### Validation of the quantitative proteomic analysis

#### SRM

To validate the results obtained by proteomic analysis, the 3 proteins with altered expression profiles were monitored by SRM in an independent group of samples (n = 10 controls, n = 10 AS patients).

**Figure 4:**
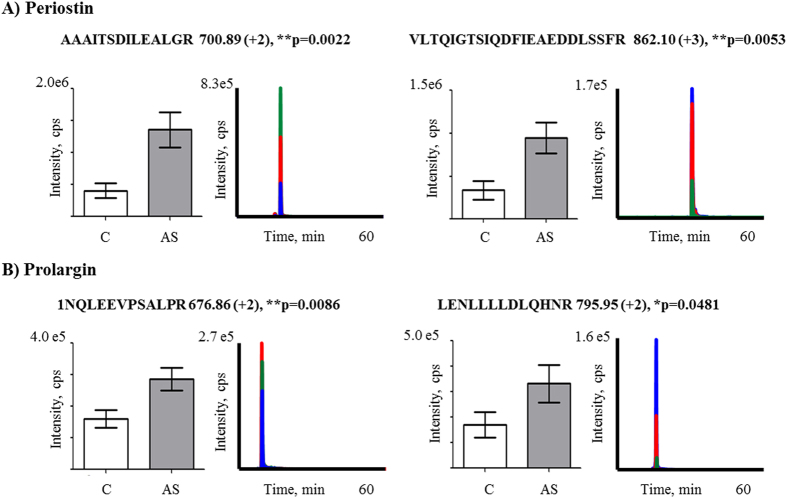
Validation of differential proteins by SRM analysis. Graphical representation of the abundance (left panels) and representative extracted ion chromatograms (right panels) of the transitions observed for periostin (**A**) and prolargin (**B**). Significant increases (p < 0.05) in the expression of the measured proteotypic peptides of both proteins were observed in AS with respect to healthy valves. *p < 0.05; **p < 0.01. C: controls; AS: aortic stenosis; cps: counts per second.

For each protein, two peptides were quantified by SRM; in the case of periostin and prolargin, these peptides were found to differ significantly in their expression between groups (Fig. 4; Panel A = periostin, peptide 1: p = 0.0022 and peptide 2: p = 0.0053 and panel B = prolargin, peptide1: p = 0.0086 and peptide 2: p = 0.0481) Unfortunately, we were not able to detect the peptides that correspond to biglycan by this technique.

#### Western blotting

**Figure 5:**
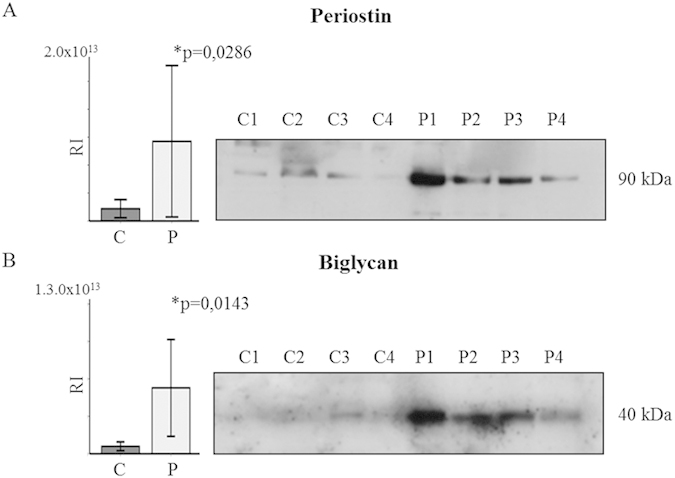
Western blot validation of the labeled proteins. (**A**) periostin (90 kDa) and (**B**) biglycan (40 kDa) levels increased in AS patients (P1 to P4) with respect the controls (C1 to C4). Corresponding p-values (Student’s t-test) for each protein analyzed are shown. *p < 0.05. RI: relative intensity.

We analyzed by WB two proteins: biglycan, not detected by SRM, and periostin with the goal in mind of confirming the reliability of both techniques. Overexpression of these proteins in AS valves compared to the control valves was confirmed (p = 0.021, p = 0.025, respectively) (Fig. 5).

## Discussion

Aortic stenosis is a progressive disease, often first manifesting as aortic valve sclerosis and later with valve dysfunction^9^. Valve dysfunction is characterized by a deregulation that depends upon the combined relationship between ECM, valve cells, and valve mechanics^3^. In fact, alterations in the normal arrangement or composition of ECM significantly and detrimentally impact valve function producing a positive feedback loop. For this reason, there has been an increasing effort to characterize the ECM proteins in the context of AS. The study of proteins of interest by proteomic approaches is an excellent tool for the identification of tissue damage associated biomarkers, which may serve to specifically identify patients with poor prognosis^10^, as well as to understand the physiological/pathological processes leading to the development of AS. Following this idea we could remark that, until now, the sole comparative proteomic analyses of AS tissue valves have been recently performed by our group^6^,^7^, with the aim of understanding the molecular mechanisms of this pathology by 2-D DIGE analysis of the aortic valve tissue and by LC-MS/MS to characterize valve secretome. Along the same line as these studies, we have identified a considerable amount of differential proteins involved in ECM processes in our study by iTRAQ, which strongly supports a deep involvement of ECM remodeling in AS pathogenesis. For this reason, and taking into account the complementarity of both techniques, we have supplemented our previous results with tissue secretome, with iTRAQ quantitative proteomics. Regarding this, we could broaden the knowledge about the disease by integrating the information related to secreted proteins with that of the molecular processes taking place inside the tissue.

With a cutoff of 1.4-fold upregulation and 0.71-fold downregulation, we could find 56 proteins to be significantly varied. Among these proteins, we found 13 proteins involved in ECM, with an outstanding cluster of 5 proteins formed by biglycan, periostin and prolargin, decorin and lumican. Of these proteins, 3 of them were validated by orthogonal techniques (WB and/or SRM). Periostin was selected for validation due to the controversy that exists in the literature about the role of this protein in AS^11^,^12^. On the other hand, biglycan and prolargin have been related to the atherosclerotic lesion. Nowadays, the relationship between AS and atherosclerosis remains unclear^13^ and therefore a deeper study of these proteins would favor to elucidate this link. Prolargin, lumican and decorin belong to the small leucine-rich proteoglycan (SLRPs) family and are closely related. Nevertheless, in contrast with lumican and decorin, the evidence about the involvement of prolargin in either atherosclerosis or AS is very limited. Besides, lumican was previously described and validated in AS patients by our group^7^.

If we focus on the molecular implications of these findings, we realize that periostin (POSTN) is a secreted protein expressed in cardiac fibroblasts, implicated in cardiac dysfunction^14^,^15^. It was first identified in bone, where it plays a key role regulating adhesion and differentiation of osteoblasts^16^,^17^. More recently, it has been suggested an involvement of POSTN in heart valve morphogenesis^18^,^19^,^20^ and cell migration^12^,^21^. Furthermore, Snider *et al*.^20^, demonstrated, that POSTN is an intriguing matrix effector protein that is required for normal ECM deposition within the heart, and suggests that POSTN may be required to enhance the fibrotic remodeling effects of TGFβ and stabilize microfibril networks within the cardiac skeleton, too. Our results showing over-expression of POSTN in stenotic valves point out a critical role of this protein in AS development, where it may contribute to small vessel formation as a result of its angiogenic activity^22^,^23^. It is important to note that this protein might enhance the penetration of microvessels into the subendothelial and fibrosa layers, which are the areas where the main anatomopathological alterations take place in all types of valvular heart disease as reported by recent studies^23^,^24^. In particular, POSTN expression was detected in the subendothelial superficial layer of normal cardiac valves and it was greatly increased in the areas of neoangiogenesis^24^ in the degenerated valves of patients with valvular heart disease, such as those with stenotic or rheumatic valves. In addition, POSTN may contribute to valve calcification via its ability to provoke osteoblast differentiation since myofibroblasts differentiate to osteoblast-like cells in severe AS valves^25^.

Regarding biglycan (BGN), a small leucine-rich proteoglycan which organizes the fibril collagen assembly^26^, it has been defined as one of the major extracellular proteoglycans found in human atherosclerotic lesions^27^,^28^. Furthermore, it is considered an early initiator of AS lesion, contributing to wall thickening^29^. Previous reports have described a positive stimulation effect of pro-inflammatory factors in the ability of macrophage cells to synthesize BGN in atherosclerosis and in hypertension^30^. Thus, BGN levels are correlated with IL-6, TNF-α, fibrinogen and CRP plasma levels, suggesting that BGN might be involved in vascular inflammation^31^. It has been recently reported that macrophage cells are able to produce BGN upon stimulation by inflammatory factors^26^. Biglycan itself has been demonstrated to modulate the immune and inflammatory responses through the interaction with Toll-Like Receptor (TLRs) signaling cascade. Therefore, during inflammation, BGN may be released to the ECM or alternatively could be induced in macrophage cells by cytokines, to signal through the interaction with TLRs. Subsequent expression of TNFα might result in increased recruitment of macrophages that release further BGN, favoring the creation of a cycle that is capable to drive an inflammatory response in both an autocrine and a paracrine manner^32^. Recently, TLRs have been detected in stenotic valves^33^, in particular in valve interstitial cells (VICs)^34^, the main cellular component of the aortic valve. Moreover, studies indicate that TLRs may play a significant role in atherosclerosis^35^,^36^. Studies with animal models have demonstrated that BGN binds to murine TLR 2 and 4, whereby it induces the production of inflammatory cytokines through the nuclear factor kappa B (NF-kB) pathway^30^. Taking into account the already postulated relationship between atherosclerosis and AS^37^ based on histological findings that point to tissue mineralization, accumulation of lipids and inflammation^38^, the observed up-regulation of BGN in AS valves found in this work is in concordance with previous studies, and suggests this proteoglycan as an early initiator of the sclerotic lesion through an interaction with TLRs within the aortic valve.

In the same way, prolargin protein (PRELP) was found up-regulated in AS valve tissue^39^,^40^. This protein is present in all types of connective tissue. As the rest of the SLRPs, PRELP has a great affinity for the collagens I and II through its leucine-rich repeat domain^41^, joins fibroblasts through its N-terminal, and plays an important role in bone remodeling control^42^. Thus, PRELP could exert a similar effect to BGN in the development of AS calcification, together with other proteoglycans such as lumican and decorin^43^,^44^, which have been also found increased in AS in the iTRAQ analysis. The increase in AS of all the SLRPs detected in the present proteomic study may serve to fundament, once again, the relationship of this pathology with atherosclerosis^36^, given that lumican, decorin and prolargin are proteins involved in both pathologies which show similar expression trends^43^.

The main limitation of our study is the low number of samples included. Despite the fact that validation was performed in an independent cohort of subjects and by two different orthogonal techniques, it would be recommendable to increase the number of patients and controls in order to confirm the potential value of the hereby described protein indicators. Moreover, a prospective study will be required to determinate the clinical utility of these proteins as potential biomarkers which could allow distinguishing patients at risk of developing degenerative AS. In conclusion, our results state the importance of the extracellular matrix remodeling in AS. The use of a comprehensive analysis of the aortic valve proteome using 2D-LC MS/MS iTRAQ methodology has allowed the identification of a specific protein profile linked to the disease, highlighting the importance of the extracellular matrix proteins. Specifically, biglycan, periostin and prolargin have been pointed out as proteins of interest and have been validated using orthogonal techniques (WB and/or SRM). These results support the existence of a dynamic extracellular matrix remodeling in human aortic valves during AS development.

## Material and Methods

### Patient and control subjects’ selection

In this work, we employed the same samples used in our previous secretome study^6^. We obtained as many subjects as possible for this kind of proteomics analysis and we eliminated any potential bias inherent in their selection which meant a lot of effort over time, mainly in control samples. Heart valves with degenerative AS (n = 18) were obtained from patients of both sexes (50% male, 50% female), with an average age of 74 ± 4 years, who underwent aortic valve replacement due to severe degenerative AS. All patients (100%) had hypertension, whereas 55% suffered hyperlipemia and 66% diabetes mellitus. Coronary artery disease was present in 55% patients and surgery was indicated according to current clinical practice guidelines. Patients with aortic regurgitation, mitral valve disease or any suspicion of rheumatic disease were not included in the study. Control valves were obtained from necropsies (n = 18). Before resecting the valve, a cardiac surgeon explored it carefully. Valves with calcifications or cusp restriction were excluded as control cases. If inserted saline in the aortic root remained in the closed valve, we ruled out significant regurgitation and we took these aortic valves as control cases. These subjects did not die from cardiovascular illnesses and had no history of coronary artery disease or diabetes mellitus (Table 3).

The study was conducted according to the recommendations of the Declaration of Helsinki and was approved by the ethics committee of Hospital Virgen de la Salud (Toledo, Spain). Signed informed consent was obtained from all subjects or relatives in case of necropsies prior to their inclusion in the study.

### Tissue Sample preparation

Aortic valves were processed according to Martin-Rojas *et al*.^6^ with minor modifications.

Briefly, AS valves were processed within a maximum of 2 hours after surgery having maintained the tissue at 4 °C in RPMI medium. The valves were washed 3 times in PBS to reduce blood contaminants. One aortic valve leaflet was then ground into powder in liquid N_2_ with a mortar and 0.2 g of this powder were resuspended in 400 μl of protein extraction buffer (10 mM Tris [pH 7.5], 500 mM NaCl, 0.1% Triton x-100, 1% β-mercaptoethanol, 1 mM PMSF)^45^,^46^. The homogenate was centrifuged at 21,000 g (5840R Eppendorf) for 15 min at 4 °C to precipitate the membranes and tissue debris, and the supernatant (E1) containing most of the soluble proteins, was collected and stored at −20 °C. The pellet was then solubilized in 7M Urea, 2M Thiourea, 4% CHAPS^47^,^48^, and centrifuged again at 21,000 g. This second supernatant (E2) was collected and the cellular debris and lipids were eliminated with the pellet. Both protein extracts (E1 and E2) were pooled, and the protein concentration was determined by the Bradford-Lowry method (Bio-Rad protein assay)^49^.

### iTRAQ labeling and SCX fractionation

iTRAQ labeling was carried out with an iTRAQ reagent 4plex Protein Quantitation kit (AB Sciex) according to manufacturer’s protocol. The amount of 100 μg of each aortic valve lysate was dissolved in 0.5 M triethylammonium bicarbonate (TEAB) (pH 8.5). Aortic valve proteins were reduced with 2 μL of (*tris*(2-carboxyethyl) phosphine) (TCEP) at 60 °C for 1 h and alkylated with 1 μL methyl methanethiosulfonate (MMTS) for 10 min at room temperature. Samples were digested using trypsin (porcine Promega) (1:40, w/w) for 16 h at 37 °C). Digested samples were further processed by labeling the derived peptides from control aortic valves with 114 and 116 iTRAQ labels, and from AS valves with 115 and 117, thus providing technical replicates within a single run (Table SM2).

The iTRAQ labeled peptides were pooled and fractionated by 2D-LC with strong cation exchange chromatography (SCX) followed by a C-18 reversed phase (RP) nano-column (100 μm I.D. and 12 cm, Mediterranea sea, Teknokroma) and analyzed in a continuous acetonitrile gradient consisting of 0–43% B in 140 min, 50−90% B in 1 min (B = 95% acetonitrile, 0.5% acetic acid). A flow rate of 300 nL/min was used to elute peptides from the RP nano-column to an emitter nanospray needle for real time ionization and peptide fragmentation on an LTQ Orbitrap XL ETD^TM^ mass spectrometer (Thermo Fisher). An enhanced FT-resolution spectrum (resolution = 60000) followed by the MS/MS spectra from most intense five parent ions were analyzed along the chromatographic run (180 min). Dynamic exclusion was set at 0.5 min. Expression changes were calculated using the reporter ion intensities (peaks areas) as control (114 + 116)/AS patients (115 + 117).

Protein identification and quantification were performed with the Protein Discoverer 1.0 software (Thermo Fisher) using MASCOT as the search engine. Two mixed cleavages were allowed, and an error of 15 ppm or 0.8 Da was set for full MS or MS/MS spectra searches, respectively. Decoy database search for FDR analysis was set at 0.05 by applying corresponding filters. Phosphorylation in Serine, Threonine, or Tyrosine was specified as variable modification due to the importance that this mechanism has in matrix remodeling^50^.

### Bioinformatics analysis of identified proteins

For a functional examination of the identified proteins, the list of the 56 significantly varied proteins was implemented on the on-line software David Bioinformatics Resources 6.7 (NIH)^51^ and Search Tool for the Retrieval of Interacting Genes/Proteins (STRING v9.1), for functional and protein interaction analyses, respectively.

Functional annotation clustering was performed in order to avoid redundancy of enriched categories and pathways (data not shown).

### Selected Reaction Monitoring (SRM)

Protein samples (10 AS patients and 10 controls) were reduced with 100 mM dithiothreitol (DTT, Sigma-Aldrich) in 50 mM ammonium bicarbonate (99% purity; Scharlau) for 30 min at 37 °C, and alkylated for 20 min at room temperature (RT) with 550 mM iodoacetamide (Sigma-Aldrich) in 50 mM ammonium bicarbonate. The proteins were then digested in 50 mM ammonium bicarbonate, 15% acetonitrile (LC-MS grade, Scharlau) with sequencing grade modified porcine trypsin at a final concentration of 1:50 (trypsin:protein). After overnight digestion at 37 °C, 2% formic acid (FA, 99.5% purity, Sigma-Aldrich) was added and the samples were cleaned with Pep-Clean spin columns (Pierce) according to the manufacturer’s instructions. Tryptic digests were dried in speed-vac and resuspended in 2% acetonitrile, 2% FA prior to MS analysis. The LC-MS/MS system consisted of a TEMPO nano LC system (Applied Biosystems) combined with a nano LC autosampler coupled to a modified triple quadrupole (4000QTRAP LC/MS/MS, Applied Biosystems). Three replicate injections (2 μg of protein in 4 μL) were performed per sample in the mobile phase A (2% ACN/98% water, 0.1% FA) at a flow rate of 10 μL/min for 5 min. Peptides were loaded onto a μ-Precolumn Cartridge (Acclaim Pep Map 100 C18, 5 μm, 100 Å; 300 μm i.d. X 5 mm, LC Packings) to preconcentrate and desalt samples. Reversed-phase liquid chromatography (RPLC) was performed on a C18 column (Onyx Monolithic C18, 150 × 0.1 mm I.D., Phenomenex) in a gradient of phase A and phase B (98% ACN/2% water, 0.1% FA). The peptides were eluted at a flow rate of 300 nL/min in a continuous acetonitrile gradient: 2–15% B for 2 min, 15–50% B for 38 min, 50 to 90% B for 2 min and 90% B for 3 min. Both the TEMPO nano LC and 4000QTRAP system were controlled by Analyst Software v.1.4.2. The mass spectrometer was set to operate in positive ion mode with ion spray voltage of 2800 V and a nanoflow interface heater temperature of 150 °C. The source and curtain gas were set to 20 and 10 psi respectively, and nitrogen was applied as both curtain and collision gas.

Theoretical SRM transitions were designed using MRMpilot software v1.1 (ABSciex), with the following settings: Enzyme = trypsin, missed cleavages = 0; modifications in peptide ≤ 3; charge states = +1 from 300 to 600 Da, +2 from 500 to 2000 Da, +3 from 900 to 3000 Da, +4 from 1600 to 4000 Da, +5 from 2400 to 10000 Da; studied modification = none; fixed modifications = carboxyamidomethylation; variable modifications = none; min. number of amino acids ≥ 5; max. number of amino acids ≤30; ignore multiple modification sites; 3 transitions per peptide (Table 4).

A pool containing a mixture of all the samples was digested as described previously and analyzed in the 4000QTrap using a MIDAS acquisition method that included the theoretical transitions. Transitions were selected when the three co-eluting peaks (corresponding to the three transitions of the same peptide) had a signal-to-noise ratio over 5 and the MS/MS data matched the theoretical spectrum for that peptide. Collision energy was optimized to obtain the maximum transmission efficiency and sensitivity for each SRM transition. A total of 12 transitions (3 per peptide) were monitored during an individual sample analysis. They were acquired at unit resolution in both Q1 and Q3, with dwell times of 50 ms resulting in cycle times of 1.8 s. The IntelliQuan algorithm included in Analyst 1.4.2 software was used to calculate the peptide abundance on the basis of peak areas after integration.

### Western blotting (WB)

Protein samples obtained from 4 AS and 4 control valves were resolved by 12% SDS-PAGE using a Miniprotean II electrophoresis unit (Bio-Rad) run at a constant current of 25 mA/gel during 1 hour. After SDS-PAGE, the proteins were transferred to a nitrocellulose membrane under a constant voltage of 15 V for 20 minutes. To ensure that equal amounts of aortic valve tissue were loaded in every polyacrylamide gel well (1-DE Western blotting) Ponceau S staining was performed on the transferred membranes. The membranes were then blocked with 7.5% non-fat milk for 1 hour^6^. Next, they were incubated overnight at 4 °C with the primary antibody followed by a 1 hour RT incubation with the specific HRP-conjugated secondary antibody, both in PBS-T containing 5% of non-fat dry milk. Detection was performed by enhanced chemiluminescence (ECL, GE Healthcare) following the manufacturers’ instructions.

The primary antibodies for Western blots included rabbit polyclonal antisera against periostin (catalog number: ab14041) and a monoclonal antibody against biglycan (catalog number: ab54855), all from Abcam (Abcam).

### Statistical analysis

Western Blot bands were measured using a GS-800 Calibrated Densitometer (Bio-Rad) and the values obtained analyzed using the statistical software packages SPSS 13.0 and GraphPad InStat (GraphPad software). A Kolmogorov-Smirnov test was applied to evaluate normal distribution of the population analyzed. When normal distribution was demonstrated, a Levene test for homogeneity of variance was performed and the Student t-test was used to compare band intensities and SRM peak areas, selecting adequate p-value in view of homogeneity results. For populations with no normal distribution, the non-parametric Mann-Whitney U test was used. For all tests, statistical significance was accepted when p < 0.05. All LC-MS/MS identifications were performed by Decoy database search for FDR analysis set at 0.05 by applying corresponding filters (Mascot Significance Threshold, and Score versus Charge state).

## Disclosures

None

## Additional Information

**How to cite this article**: Martin-Rojas, T. *et al*. iTRAQ proteomic analysis of extracellular matrix remodeling in aortic valve disease. *Sci. Rep*. **5**, 17290; doi: 10.1038/srep17290 (2015).

## Supplementary Material

Supplementary Information

## Figures and Tables

**Table 1:** Proteins differentially expressed (upregulated in AS) and present in the four replicates from either of the two groups after 2D-LC iTRAQ analysis with a fold change <0.71 (RatioControl/AS).

**Protein Name**	**Accession Number**	**Ratio**	**Function**
Histone H2A type 1-H	gi 18105045	0.662	Complex assembly
Serotransferrin precursor	gi 4557871	0.628	EC region/matrix; Inflammatory/immune response; Transport; Homeostasis
Protein Muted homolog isoform 1	gi 41152237	0.606	Homeostasis
Serum albumin preproprotein	gi 4502027	0.603	EC region/matrix; Oxidative stress; Transport; Homeostasis; Vesicles
Calcineurin-binding protein cabin-1 isoform a	gi 6912458	0.600	Enzymatic activity
Superoxide dismutase [Mn], mitochondrial	gi 67782309	0.561	Oxidative stress; Inflammatory/immune response; Transport; Homeostasis; Glucose metabolism; Complex assembly
Phosphoglycerate kinase 1	gi 4505763	0.550	Glucose metabolism
Alpha-1-antitrypsin	gi 50363221	0.534	EC región/matrix; Oxidative stress; Infammatory/immune response; Enzymatic activity; Homeostasis; Vesicles
Serum amyloid P-component	gi 4502133	0.478	EC region/matrix; Inflammatory/immune response; Binding; Complex assembly
Annexin A1	gi 4502101	0.660	Inflammatory/immune response; Cytoskeleton binding protein; Enzymatic activity; Homeostasis
Glutathione peroxidase 3	gi 6006001	0.577	EC region/matrix; Oxidative stress; Homeostasis; Complex assembly
Collagen alpha-1(VI) chain	gi 87196339	0.663	EC región/matrix
Beta-galactoside-binding lectin L-14-I	gi 4504981	0.659	EC region/matrix; Cytoskeleton organization; Homeostasis; Binding
Alpha-enolase	gi 4503571	0.650	Muscle related protein; Transport; Glucose metabolism
L-lactate dehydrogenase A chain	gi 5031857	0.650	Glucose metabolism
Triosephosphate isomerase isoform 1	gi 4507645	0.647	Oxidative stress; Homeostasis; Glucose metabolism
Moesin	gi 4505257	0.644	Cytoskeleton protein binding
Peroxiredoxin-1	gi 4505591	0.644	Oxidative stress; Inflammation/immune response; Homeostasis; Glucose metabolism
Peptidyl-prolyl cis-trans isomerase A	gi 10863927	0.629	Cytoskeleton protein binding
Similar to Ig gamma-1 chain C region	gi 89037890	0.627	Homeostasis
Transforming growth factor-beta-induced protein ig-h3	gi 4507467	0.624	EC región/matrix
Protein S100-A6	gi 7657532	0.597	Cell morphogenesis; Ion binding (NC)
Superoxide dismutase [Cu-Zn]	gi 4507149	0.581	EC región/matrix; Oxidative stress; Inflammation/immune response; Muscle related protein; Cytoskeleton protein binding; Cytoskeleton organization; Homeostasis; Vesicles
Inter-alpha (globulin) inhibitor H1	gi 4504781	0.567	EC región/matrix; Enzymatic activity
Vimentin	gi 62414289	0.586	Cytoskeleton protein binding
Prolargin	gi 4506041	0.594	EC región/matrix; Binding
Tubulin beta chain	gi 29788785	0.610	Cytoskeleton protein binding; Complex assembly
Alcohol dehydrogenase 1B	gi 34577061	0.592	Oxidation/reduction (NC)
Annexin A2	gi 4757756	0.584	EC region/matrix; Cytoskeleton protein binding; Enzymatic activity; Vesicles
Endoplasmin precursor	gi 4507677	0.532	Homeostasis; Vesicles
Periostin, osteoblast specific factor	gi 5453834	0.550	EC region/matrix; Oxidative stress; Binding
Biglycan	gi 4502403	0.701	EC región/matrix; Oxidative stress; Vesicles; Binding
Decorin isoform a preproprotein	gi 4503271	0.610	EC región/matrix; Oxidative stress; Binding
Hemopexin	gi 11321561	0.630	EC region/matrix; Oxidative stress; Transport; Homeostasis
Lumican	gi 4505047	0.582	EC región/matrix
Haptoglobin	gi 4826762	0.519	EC region/matrix; Inflammatory/immune response; Homeostasis
Orosomucoid 1	gi 9257232	0.515	EC region/matrix; Inflammatory/immune response

**Table 2:** Proteins differentially expressed (downregulated in AS) and present in the four mixtures from either of the two groups after 2D-LC iTRAQ analysis with a fold change >1.4 (Ratio Control/AS).

**Protein Name**	**Accession Number**	**Ratio**	**Function**
Calponin-1	gi 21361120	3.035	Cytoskeleton protein binding; Cytoskeleton organization
Tropomyosin beta chain isoform 2	gi 47519616	2.408	Muscle related protein; Cytoskeleton protein binding; Cytoskeleton organization
Alpha-2-globin	gi 4504345	2.323	Transport
Transgelin	gi 48255905	2.219	Muscle related protein; Cytoskeleton binding protein
Myosin light polypeptide 6	gi 88999583	2.100	Muscle related protein; Cytoskeleton binding protein; Cytoskeleton organization
Tropomyosin alpha	gi 63252902	1.868	Oxidative stress; Inflammation/immune response; Muscle related; Cytoskeleton protein binding; Cytoskeleton organization
Beta-1-globin	gi 4504349	1.784	Oxidative stress; Transport
Haptoglobin	gi 4826762	1.694	EC region/matrix; Inflammatory/immune response; Homeostasis
Delta-globin	gi 4504351	1.581	Transport
Myosin-11 isoform SM2A	gi 13124875	1.574	EC region/matrix; Muscle related protein; Cytoskeleton protein binding; Cytoskeleton organization; Vesicles; Complex assembly
Alpha-2-HS-glycoprotein	gi 4502005	1.643	EC region/matrix; Inflammatory/immune response; Enzymatic activity
Actin, gamma-enteric smooth muscle isoform 1	gi 4501889	2.351	Oxidative stress; Muscle related protein; Cytoskeleton protein binding; Cytoskeleton organization
Apolipoprotein C-I	gi 4502157	1.510	EC region/matrix; Enzymatic activity; Complex assembly
Filamin-1	gi 4503745	1.860	EC region/matrix; Muscle related protein; Cytoskeleton protein binding; Cytoskeleton organization; Complex assembly
Myosin-9	gi 12667788	1.830	Inflammation/immune response; Cytoskeleton protein binding; Cytoskeleton organization
Transgelin-2	gi 4507357	1.756	Muscle related protein
Milk fat globule-EGF factor 8 protein	gi 5174557	1.682	EC region/matrix; Enzymatic activity
Peptidyl-prolyl cis-trans isomerase A	gi 10863927	1.639	Cytoskeleton protein binding
L-lactate dehydrogenase A chain isoform 1	gi 5031857	1.602	Glucose metabolism
Profilin-1	gi 4826898	1.589	Muscle related protein; Cytoskeleton protein binding; Cytoskeleton organization
Four and a half LIM domains protein 1 isoform 2	gi 21361122	1.521	Muscle related protein
Zinc finger protein 648	gi 58000461	1.529	Regulation of transcription (NC)

**Table 3:** Clinical characteristics.

	**AS (n = 18)**	**C (n = 18)**
Age	74.4 ± 4	61 ± 16
Sex (%Male/Female)	50/50	83/17
% AHT	100	17
% Dyslipemia	55	—
% Diabetes	66	0
% Coronary disease	55	0

The same samples that were used in our previous secretome study were employed in this work^6^. (AHT) arterial hypertension.

**Table 4:** List of proteins monitored by SRM analysis.

**Protein**	**Accession number**	**Peptide sequence**	**Q1**	**Q3 (fragment ion)**		
				**T1**	**T2**	**T3**
Periostin	gi 5453834	AAAITSDILEALGR	700.89	886.5	973.53	1074.58
		VLTQIGTSIQDFIEAEDDLSSFR	862.10	1039.47	1168.51	1281.6
Prolargin	gi 4506041	NQLEEVPSALPR	676.86	868.49	997.53	1110.62
		LENLLLLDLQHNR	795.95	1008.56	1121.64	1234.73
