# Supplementary material for: iTRAQ proteomic analysis of extracellular matrix remodeling in aortic valve disease
Source: Sci Rep. 2015 Dec 1;5:17290. doi: 10.1038/srep17290 (PMC4664895; doi:10.1038/srep17290)
Supplement: Supplementary Information [file srep17290-s1.pdf]

iTRAQ proteomic analysis of extracellular matrix remodeling in aortic valve disease

Tatiana Martin-Rojas[1†], Laura Mourino-Alvarez[1†], Sergio Alonso-Orgaz[1], Esther Rosello-Lleti[2], Enrique Calvo[3], Luis Fernando Lopez-Almodovar[4], Miguel Rivera[2], Luis R. Padial[5], Juan Antonio Lopez[3], Fernando de la Cuesta[1], Maria G. Barderas[1*]

[1]*Department of Vascular Physiopathology, Hospital Nacional de Parapléjicos, SESCAM, Toledo, Spain.*

[2]*Cardiocirculatory Unit, Health Research Institute, Hospital La Fe, Valencia, Spain.*

[3]*Proteomics Core Facility, CNIC, Madrid, Spain.*

[4]*Cardiac Surgery, Hospital Virgen de la Salud, SESCAM, Toledo, Spain.*

[5]*Department of Cardiology, Hospital Virgen de la Salud, SESCAM, Toledo, Spain.*

† Both authors contributed equally to this work.

Corresponding author: M.G. Barderas, Laboratorio de Fisiopatologia Vascular, Edificio de Terapia 2ª planta, Hospital Nacional de Paraplejicos, SESCAM, 45071 Toledo, España. e-mail: megonzalezb@sescam.jccm.es FAX: 0034925247745. PHONE: 0034925396826

Figure SM1. iTRAQ analysis workflow. Schematic representation of the workflow and the subsequent iTRAQ analysis for aortic valve tissue showing sample preparation, protein extraction, iTRAQ labeling, LC-MS/MS analysis and results validation.

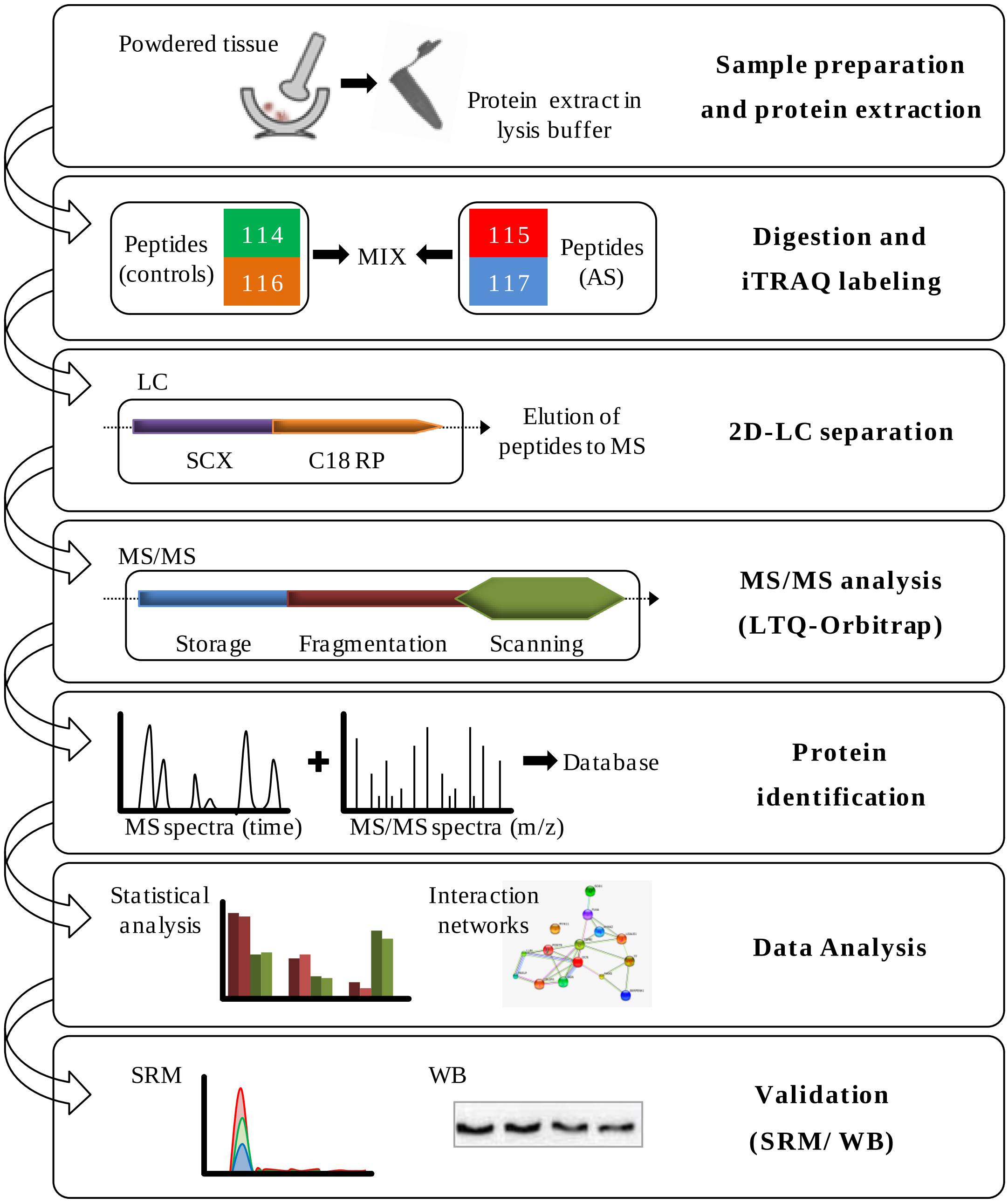

Table SM1. Pathway analysis revealed a significant enrichment in glycolysis pathway (both BIOCARTA and KEGG).

Category	Term	Count	P-Value	Fold Enrichment
KEGG	Glycolysis/Gluconeogenesis	5	0.00013	17.66
Biocarta	Glycolysis Pathway	3	0.00282	32.66

Table SM2. Experimental design of iTRAQ isobaric labeling. Controls samples were labeled with 114 and 116 tags, while AS valves were labeled with 115 and 117 tags.

iTRAQ 114	iTRAQ 116	iTRAQ 115	iTRAQ 117	MIXTURES
Control 1	Control 2	Patient 1	Patient 2	Mix 1
Control 3	Control 4	Patient 3	Patient 4	Mix 2
Control 1	Control 2	Patient 3	Patient 4	Mix 3
Control 3	Control 4	Patient 1	Patient 2	Mix 4
